# Data on the peptide mapping and MS identification for phosphorylated peptide

**DOI:** 10.1016/j.dib.2016.05.009

**Published:** 2016-05-12

**Authors:** Hui Wang, Zong-cai Tu, Guang-xian Liu, Lu Zhang, Yuan Chen

**Affiliations:** a State Key Laboratory of Food Science and Technology, Nanchang University, Nanchang, Jiangxi, China; b College of Life Sciences, Jiangxi Normal University, Nanchang, Jiangxi, China

## Abstract

This article contains peptides mapping, mass spectrometry and processed data related to the research “Identification and quantification of the phosphorylated ovalbumin by high resolution mass spectrometry under dry-heating treatment” [1]. Fourier transform ion cyclotron mass spectrometry (FTICR MS) was used to investigate the specific phosphorylation sites and the degree of phosphorylation (DSP) at each site. Specifically, phosphorylated peptides were monitored through mass shift on the FTICR MS spectrum. DSP was evaluated through the relative abundance levels of the FTICR MS spectrometry. From these data, the calculation method of DSP was exemplified.

**Specifications Table**

**Table:** 

Subject area	Chemistry, Biology
More specific subject area	Mass spectrometric analysis of the phosphorylation sites and degree
Type of data	Table, figures
How data was acquired	Mass spectrometry data were collected on FTDoc Viewer
Data format	Analyzed
Experimental factors	Phosphorylated ovalbumin under dry-heating at 85 °C for 1, 2, and 5 days. Pepsin was used to digest the protein
Experimental features	Identification of the phosphorylation sites and degree of the phosphorylation
Data source location	Nanchang University, Nanchang, China
Data accessibility	Data is provided within this article

**Value of the data**

• Precise mass shift in FTICR Mass Spectrum could characterize of the phosphorylated protein.
• The abundance of the phosphorylated peptide was used to calculate the degree of phosphorylation (DSP) at each site.
• Method to calculate the DSP from the mass peak abundance.

## 1 Data

**Fig. 1:**
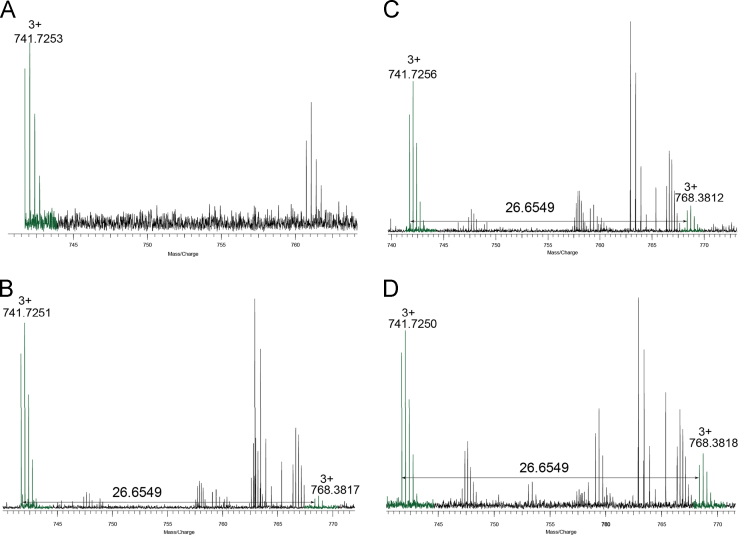
FTICR MS of peptide 366–385 (^366^FCIKHIATNAVLFFGRC VSP^385^) at *m*/*z* 741.72^3+^ from natural Oval (A) and P-Oval incubated for 1 day (B), 2 days (C), and 5 days (D). Phosphorylation is indicated by a mass increase of 79.9663 Da.

**Fig. 2:**
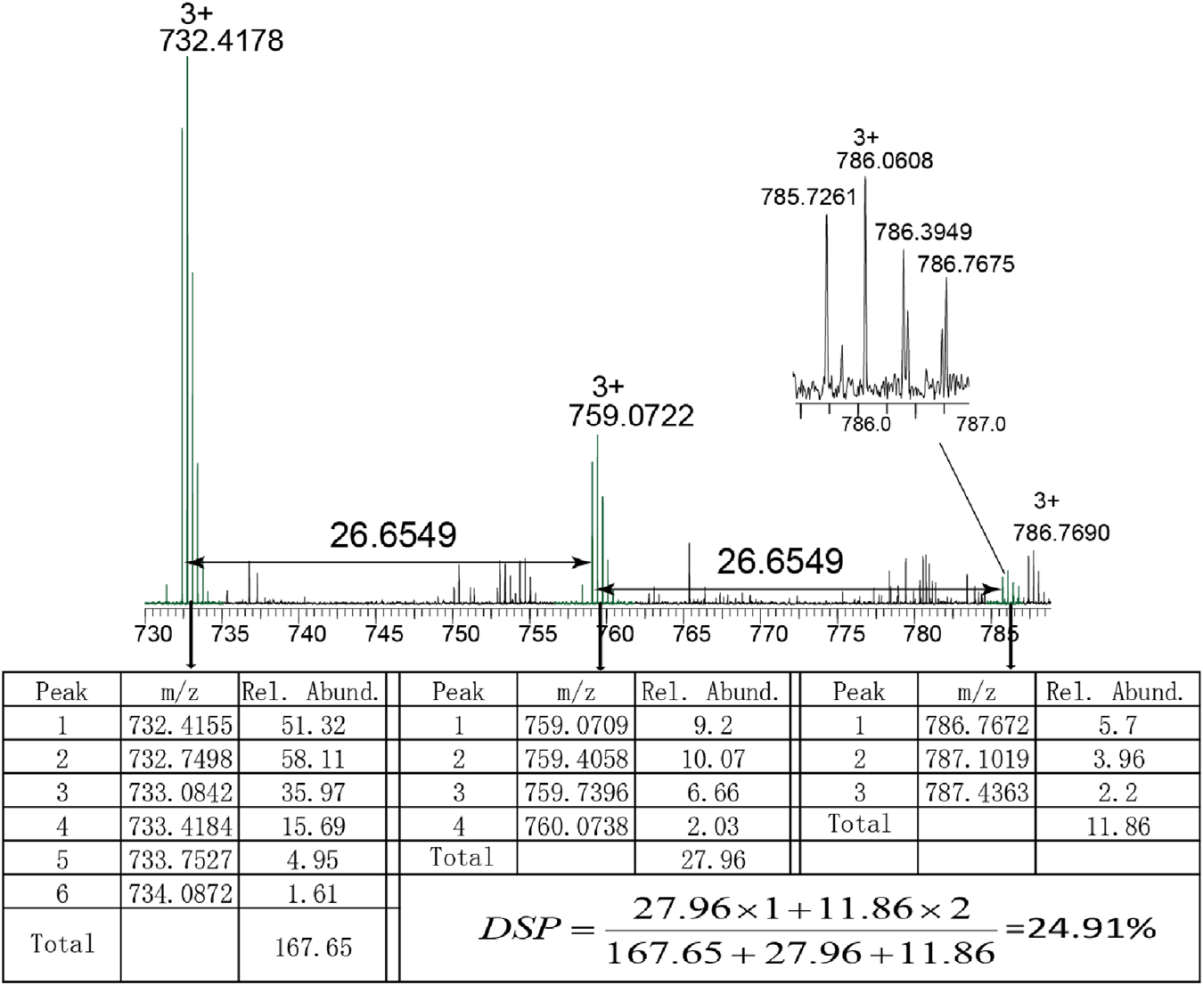
DSP calculation of peptide 41**–**59 with *m*/*z* of 732.41783^+^ after 1 day of incubation.

The data include table and figures, which help analyze the phosphorylated peptides and DSP. The peptic peptides detected by LC FTICR MS after 5 min digestion of natural ovalbumin as control are listed in Supplementary Table 1. Compared with the control, the phosphorylated peptides were directly determined from the mass increases of 79.9663 or its multiples. From these data, we exemplified the mass spectrometry to identify and calculate the DSP of each phosphorylated peptide (Figs. S1–S3 and Fig. 1). Here, we also exemplified the DSP calculation method from the peak abundance (Fig. 2).

## 2 Experimental design, materials and methods

### 2.1 Chemicals and preparation of the phosphorylated ovalbumin (P-Oval)

Ovalbumin (Grade V, A-5503) was purchased from Sigma-Aldrich (St. Louis, MO, USA). Ovalbumin was dissolved in 0.1 M sodium pyrophosphate buffer, and then lyophilized and incubated at 85 °C for 1, 2, and 5 days, respectively [2].

### 2.2 FTICR MS

The P-Oval was dissolved, and free sodium pyrophosphate was removed by zip-tip. Pepsin was used to digest the samples. Peptides were identified through HPLC FTICR MS match with the accurate masses through *Protein Prospector Tools*.

### 2.3 Peptide mapping

From these data, pepsin was used to cut the protein at defined sites to generate small peptides subjected to measure the peptide mass. The peptic peptides were detected by LC FTICR MS after 5 min of digestion of natural ovalbumin. (Supplementary Table 1)

### 2.4 Identification of the phosphorylated peptide

Enrichment of the phosphorylated peptides prior to mass spectrometric analysis is often required [3]. Calculating the phosphorus content of each phosphorylated peptide because of different retention times is difficult. From these data, no enrichment was performed to the peptide, and its phosphorylated peptide was eluted at the same retention time. A peptide is phosphorylated by one phosphate, the corresponding *m*/*z* peaks with one, two, and three charges will display *m*/*z* increases of 79.9663, 39.9826 and 26.6549, respectively [4]. For the dual- and tri-phosphorylated peptides, the mass increases should be equal to 159.9327 and 239.8990 Da, respectively.

Figs. S1–S3 and Fig. 1 show the mass spectra of four peptic peptides from natural ovalbumin and P-Oval incubated for 1, 2, and 5 days. For peptides 366**–**385 (*m*/*z* 741.72513^+^), a peak with *m*/*z* of 768.38173^+^ was emerged after 1 day of incubation with increasing abundance after 2 and 5 days (Fig. 1). The *m*/*z* difference of these two peaks was 26.6564, equivalent to a mass shift of 79.9695 Da, indicating that this peptide was modified by one molecular equivalent of HPO_3_.

For peptides 41**–**59 (*m*/*z* of 732.41603^+^ and *m*/*z* of 921.46533^+^), peaks with mass shifts of 79.9677 and 79.9668 (*m*/*z* of 759.07253^+^ and 948.12313^+^), respectively, appeared after 1 day of incubation (Figs. S1 and S2). The intensity of the phosphorylated form (759.07253^+^ and 948.12313^+^) was increased after 2 and 5 days of incubation. In addition, a third peak with *m*/*z* of 785.72613^+^ and 974.77823^+^ emerged. Compared with the mono-phosphorylated peak, the newly emerged peaks underwent a further *m*/*z* increase shift of 26.6564 (equivalent to a mass shift of 79.9695 Da), indicating that these two peptides were modified by an additional phosphate molecule. Thus, two molecules of phosphate were added to these two peptides to form dual-phosphorylated peptides after 2 and 5 days of dry heating. No tri-phosphorylated form of this peptide was found. However, the intensity of the mono-phosphorylated and dual-phosphorylated forms was further increased (Figs. S1 and S2).

Similarly, Fig. S3 provides another example for a peptide with two charges. Peptide 142**–**157 with *m*/*z* of 929.99102^+^ exhibited a *m*/*z* increase of 39.9826 (equivalent to a mass increase of 79.9695 Da), after 1 day of incubation, indicating that mono-phosphorylated of this peptide occurred. The dual-phosphorylated form of this peptide was present after 2 and 5 days of incubation.

### 2.5 Calculation of the phosphorus content

In mass spectrum, peptide and its phosphorylated peptide were detected at the same retention time. Therefore, the average degree of substitution per peptide (DSP) can be calculated through the relative abundance according to the following formula:

$$DSP=\frac{\sum_{i=0}^{n}i\times I(peptide+i\times phosphorus)}{\sum_{i=0}^{n}I(peptide+i\times phosphorus)}$$

where *I* is the sum of the intensities of every P-Oval peptide, and *i* is the number of phosphorus units attached to the peptide.

From these data, we exemplified the DSP calculation method by the peptide 41**–**59 with *m*/*z* of 732.41603^+^ after 1 day of incubation. As shown in Fig. 2, DSP was calculated through the relative abundance of peptide and phosphorylated peptides in one mass spectrum. The mass peaks of the peptide and its phosphorylated peptides appeared at the same retention time which could ensure that the relative phosphorylation degree is closed to the actual value.
